# Bilateral high origin and superficial trajectory of the deep femoral artery: clinical and applied anatomy

**DOI:** 10.4322/acr.2024.492

**Published:** 2024-06-07

**Authors:** Gabriel Deveaux, William P. Mayer

**Affiliations:** 1 Dalhousie University, Dalhousie Medicine New Brunswick, Saint John, NB, Canada

**Keywords:** Anatomy, Regional, Anatomic Variation, Femoral Artery, Dissection, Case Reports

## Abstract

The anatomy of the femoral triangle is explored in various approaches, ranging from pulse verification to invasive catheterization procedures. Within the femoral triangle, the deep femoral artery is one of the vessels reported to present several anatomical variations that must be considered before clinical or surgical interventions. Here, we are reporting a unique bilateral variation of the deep femoral artery for medical education purposes and reflecting on its applied, surgical, and clinical anatomy. During the dissection of the femoral triangle, we observed that the deep femoral artery originated in the vicinity of the inguinal ligament and ran in parallel with the femoral artery in a superficial trajectory on both sides of the donor. On the right side, the DFA continued superficial for 8.8 cm, with an origin of 1.2 cm inferior to the inguinal ligament. On the left side, it presented a similar anatomical arrangement, though with an origin of 1.6cm inferior to the inguinal ligament and a superficial course of 5cm. The position of the lateral circumflex femoral vein posterior to the deep femoral artery played a role in this distinctive, lengthy, and superficial presentation of the deep femoral artery. This anatomical variation directly affects surgical procedures, diagnostics, and endovascular interventions. A deep femoral artery with such a lengthy superficial trajectory can be mistakenly used for catheterization instead of the femoral artery or be injured, disrupting the main blood supply of the thigh muscles.

## INTRODUCTION

The femoral artery (FA) is the direct continuation of the external iliac artery. In the upper third of the thigh, its trajectory remains relatively superficial within the femoral triangle, where it is accessible for pulse verification or catheterization procedures. The FA extends from the inguinal ligament to the adductor hiatus and courses within the adductor canal before becoming the popliteal artery. Typically, it gives rise to the following branches as it descends within the femoral triangle: 1- Superficial epigastric artery, 2- Superficial circumflex iliac artery, 3- Superficial external pudendal artery, 4- Deep external pudendal artery, 5- Deep femoral artery and 6- Descending genicular artery.^1-3^

The deep femoral artery (DFA) is the largest FA branch and the thigh’s main supply. It typically arises as a lateral branch of the FA, 3-4 cm distal to the inguinal ligament, and immediately dives deep in relation to the FA within the femoral triangle. The DFA gives rise to the medial and lateral circumflex femoral arteries (MCFA and LCFA), then descends along the medial aspect of the femur, resting on the anterior surface of the adductor brevis and magnus muscles. Along its final course, the DFA often originates 3 to 4 perforating femoral arteries that pierce the adductor magnus, winding around the femur to supply the various thigh compartments.^4^

The basic anatomy of the DFA is relatively consistent, although some varieties have been reported since the nineteenth century,^2^ and these include anatomical variations in its branching patterns^5^ or trajectory within the femoral triangle.^6^ Anatomical variation and meta-analysis studies have reported six different patterns of DFA origin in the proximal 1/3 of the thigh, and several variations of the MCFA and LCFA.^7,8^ Usually, these vessels play crucial roles in the vascular supply to the thigh, hip, femoral neck, and surrounding structures. MCFA and LCFA variants may arise directly from the FA,^9,10^ which in turn makes the DFA originate in the middle 1/3 of the thigh with a significantly smaller caliber.^7^

In this manuscript, we are reporting a unique case of bilateral high origin associated with a superficial trajectory of the DFA and reflecting on its clinical implications for diagnostics, surgical procedures, and endovascular interventions, with the goal that dissemination of this rare case will be beneficial not only for medical education purposes but also to planning, anticipation, and prevention of iatrogenic problems during anterior femoral region interventions.

## CASE REPORT

This case was observed in a 75-year-old white female body, whose cause of death was recorded as ventricular arrest. The individual donated and dedicated their body to medical education and research by providing premortem informed consent to the Dalhousie University Human Body Donation Program. After the admittance of the donor, the dissection of the femoral triangle was performed as part of hands-on training offered in a clinical and applied anatomy elective course. A four-layer dissection of the region was executed following: 1- skin, 2- subcutaneous tissue, 3- muscular fascia and superficial boundaries of the femoral triangle, and 4- deep region and content of the femoral triangle. During the dissection, we detected that the DFA originated close to the inguinal ligament and ran in parallel with the FA in a superficial trajectory in the roof of the femoral triangle on both sides of the donor.

## AUTOPSY PRESENTATION

**Figure 1:**
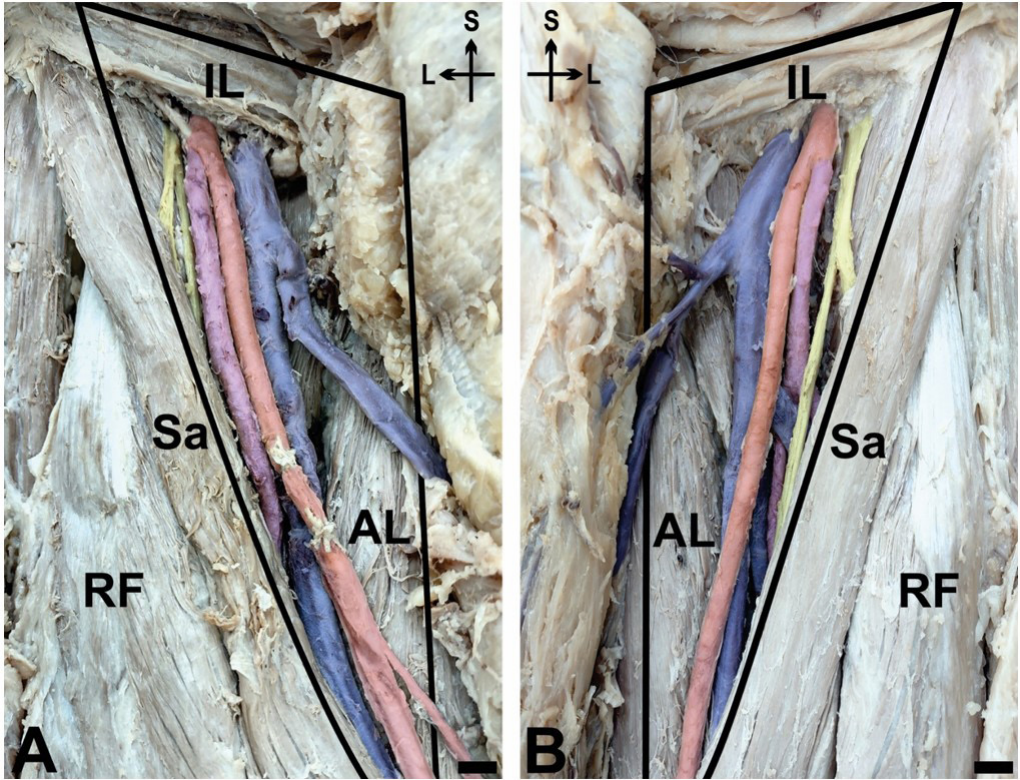
Bilateral high origin and superficial trajectory of the deep femoral artery. **A** and **B** – right and left superficial dissections of the femoral triangle. *IL* = inguinal ligament; *Sa* = sartorius; *AL* = adductor longus; *RF* = rectus femoris; *Blue* = femoral vein and its tributaries; *Red* = femoral artery; *Magenta* = deep femoral artery; *Yellow* = femoral nerve; *S* = superior; *L* = lateral. Scale bar 1cm.

During dissection of the inguinal region and the femoral triangle, we observed that the DFA originated in the vicinity of the inguinal ligament, running in parallel with the FA on both sides. On the right side of the body (Figure 1A), the DFA originated 1.2cm inferiorly to the inguinal ligament. Its initial trajectory was lateral and parallel to the FA, remaining superficial for 8.8cm. During its superficial course, the DFA was bundled with the femoral vein, FA, and femoral nerve within the femoral sheath. The DFA on the left side of the body (Figure 1B) presented a similar anatomical arrangement, though with an origin of 1.6cm inferior to the inguinal ligament and a superficial course of 5cm.

**Figure 2:**
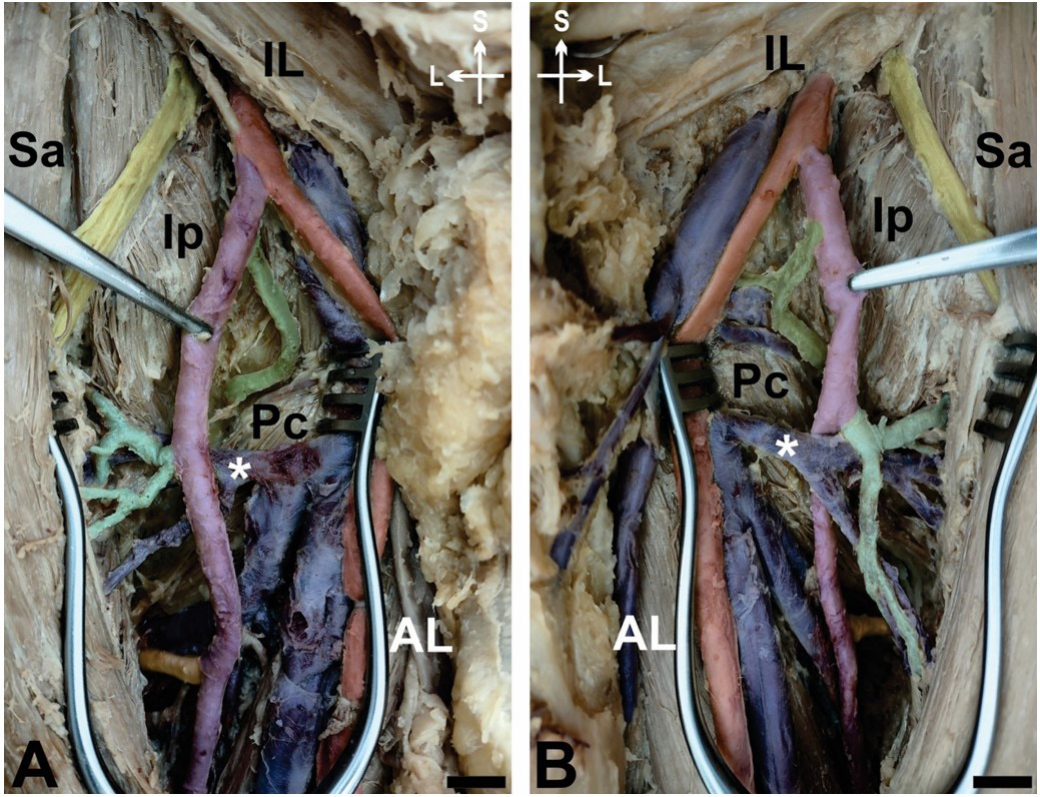
Deep femoral artery and its branches. **A** and **B** – right and left deep dissections of the femoral triangle, superficial structures within the femoral triangle were retracted to show the course of the deep femoral artery. *IL* = inguinal ligament; *Sa* = sartorius; *AL* = adductor longus; *Ip* = iliopsoas; *Pc* = pectineus; *Blue* = femoral vein and its tributaries; *Red* = femoral artery; *Yellow* = femoral nerve; *Magenta* = deep femoral artery; *Green* = medial circumflex femoral artery; *Cyan* = lateral circumflex femoral artery; *lateral circumflex femoral vein with different arrangements related to the deep femoral artery on both thighs; *Orange* = first perforating femoral artery; *S* = superior; *L* = lateral. Scale bar 1cm.

Furthermore, the superficial portion of the DFA and the FA exhibited the same diameter ipsilaterally, 0.7cm on the right side and 0.6cm on the left thigh. The branches of the DFA were present and typically arranged within the deep region of the femoral triangle on both thighs; however, we noticed that the lateral circumflex femoral vein crossed the DFA on the right side posteriorly (Figure 2A), in contrast with an anterior crossing observed on the left side (Figure 2B).

## DISCUSSION

The DFA, also known as the arteria femoris profunda, is a major branch of the FA. Although various labels have been used to identify this vessel,^4,5^ we have adopted the International Anatomical Terminology^1^ instead of any clinical or alternative nomenclature.

The main anatomical variations of the DFA reported in the literature are cataloged within the following groups: 1- *high origin*,^2,6,9,11,12^ cases in which the DFA originates in the pelvis or in 1-2 cm inferiorly to the inguinal ligament; 2- *accessory DFA*,^11,13^ an additional branch that arises near or isolated from the main DFA and follows a separate course; 3- *absence or hypoplasia*,^14-16^ DFA is missing or significantly reduced, in such cases the blood supply is maintained through collateral anastomoses; 4- *branching variations*,^3,11,12,17^ the number, size, origin, and branching pattern of the medial and lateral femoral circumflex arteries as well as perforating arteries differ in a variety of ways; 5- *course of the DFA*,^6,7,12,17^ the main stem of the DFA is presented in a lateral, posterolateral, posterior, posteromedial or medial arrangement in relation to the FA.

Our case report shows a unique and unreported combination of two of these variations: high origin and unusual superficial lengthy course of the DFA. Noteworthy is that the DFA reported here ran superficially and in parallel with the femoral artery in the roof of the femoral triangle to a considerable extent. Interestingly, this superficial trajectory of the DFA was lengthier in the right thigh. The position of the lateral circumflex femoral vein posterior to the DFA may have played a role in this superficial presentation and may characterize a novel observation among vascular variations within the femoral triangle. Although the left DFA had an initial superficial trajectory as well, it dove to a deeper region of the femoral triangle as soon as the lateral circumflex femoral vein crossed its anterior surface (asterisk Figures 2A and 2B). We could not find a similar report of the DFA and its superficial path related to the lateral circumflex femoral vein in an extensive literature review. Nonetheless, the literature described 2 rare cases in which the DFA was bounded by the femoral vein medially before entering its distribution area, and this arrangement possibly contributes to phlebothrombosis of the femoral vein.^18^

The lower limb vascular embryology is a fascinating and complex area of study. The ventral (femoral) and dorsal (sciatic) branches of the fifth embryological lumbar artery anastomose to form the lower limb arteries.^19^ In a later stage, the sciatic artery regresses as it is transiently incorporated into the region of the future popliteal artery; during this process, the connection of both embryologic vessels is severed, and the posterior thigh becomes supplied by the deep femoral artery originated from the embryologic ventral femoral vessel.^14^ This vascular development is dependent on molecular mechanisms that are not completely understood, and subtle deviations in these processes can lead to variations in vessel growth and patterning.^14^ It is likely that the asymmetry found in the lateral circumflex femoral vein pushing the DFA to a more extensive superficial trajectory on the right thigh is derived from those complex mechanisms beyond the scope of this case report.

It is important to note that this anatomic variation has direct implications for surgical procedures, diagnostic, and endovascular interventions involving the lower limb, abdomen, thorax, head, and neck. A DFA with such a superficial trajectory can be mistakenly used for catheterization instead of the FA. A tangible example is given by any procedure where the FA must be canulated caudally to assess the morphology and integrity of the popliteal artery and its terminal branches. Certainly, caudal canulation of the DFA would never give access to the popliteal artery and its distal branches distributed to the leg. On the other hand, if the DFA with such a variation is cannulated cranially for assessment of upper arterial lines of the body (e.g., abdominal vessels, heart chambers, coronaries, great vessels, etc.) the intervention may be successful due to the caliber and parallel arrangement of the DFA with the FA presented in this case; however, studies show that puncture of the DFA may latter develop into pseudoaneurysms.^20,21^

Additionally, complications after hip arthroscopy, arthrodesis, and internal fixation operations include pseudoaneurysms of the DFA.^22^ A high and superficial DFA, as reported here, may be in a critical position during an anterior (Smith-Petersen) or anteromedial (Weinstein) approaches to the hip. The DFA in such a superficial trajectory may be subjected to undesirable surgical manipulation. Although most of the DFA pseudoaneurysms develop after surgical procedures, femur fractures, or penetrating wounds, there are reports of DFA pseudoaneurysms arising after blunt trauma without association with femur fracture.^23^ A variation such as the one reported herein is less guarded by the sartorius and rectus femoris muscles and may be more vulnerable to blunt trauma in the femoral triangle.

Awareness of variations such as the one reported here is crucial, given that the FA and the DFA are accessed in a variety of procedures such as embalming, arteriography, hemodialysis, reconstructive surgeries, and angioplasty.^19^ Point-of-care ultrasonography (PoCUS) study has become an integral part of medical education.^24^ Particularly in the femoral triangle, PoCUS may be used to guide the placement of central venous catheter, arterial lines, or femoral nerve block. A PoCUS transverse window of the femoral artery in the femoral triangle with this variation, is likely to show 2 arteries (DFA and FA) arranged superolateral to the femoral vein, an atypical organization compared to the common arrangement of FA superior to the DFA, with both displayed lateral to the femoral vein.

Given the complexity of variations in this area, a careful imaging analysis of the femoral triangle and subinguinal region should be considered not only to plan but also to avoid intra- and post-surgical complications in cases of DFA anatomical variations. We trust that clinical discussion and dissemination of this rare case add value to medical education literature and is beneficial to the planning, anticipation, and prevention of iatrogenic problems during anterior femoral region interventions.

## CONCLUSION

This report describes a rare anatomical variation of the deep femoral artery and deliberates on its applied, surgical, and clinical anatomy. A deep femoral artery with such a high origin and superficial trajectory might be mistakenly used for percutaneous cannulation. The deep femoral artery in this report is less guarded by the sartorius and rectus femoris muscles and may be more vulnerable to blunt trauma in the femoral triangle.
